# Basic and clinical immunology – 3029. Secretory leukocyte protease inhibitor plays an important role in the regulation of eye inflammation and wound healing

**DOI:** 10.1186/1939-4551-6-S1-P204

**Published:** 2013-04-23

**Authors:** Andrés Ricardo Grenat, Victor Eduardo Reviglio, Ruben Horario Sambuelli

**Affiliations:** 1 Physiology, Experimental Eye Pathology, Facultad De Medicina Universidad Católica De Córdoba, Córdoba, Argentina

## Background

We undertook this study to investigate the expression of SLPI in ocular tissues and to determine its role as an antimicrobial and antiprotease agent.

## Methods

We used immunohistochemistry to localize SLPI and MMPs in normal and inflamed-infected rat eye tissues. The expression levels of SLPI mRNA protein were evaluated by RT-PCR and western blotting. Eighty female Lewis rats (200g) were randomly divided into four test groups as follows: 1) Staphylococcus aureus endophthalmitis, 2) S.a. keratitis, 3) HSV keratitis, 4) Photorefractive keratectomy. Forty eight hours post-injury or infection, the animals were sacrificed and the eyes processed for the experimental studies. The values of SLPI bands from western blots were quantified by densitometry analysis and subjected to statistical analysis (p 0.05, Mann-Whitney test) to determine whether there was a difference between the treatment and the control eye groups.

## Results

Eyes in the control group did not reveal inflammatory changes at slit lamp examination or in histopathology studies. When examined for presence of SLPI, none of eyes studied from the control group had any discernible immunohistochemical reaction. However in the treatment groups, immunohistochemical staining for SLPI and MMPs showed that SLPI is expressed mainly in the inflamed and compromised tissues associated to inflammatory cells recruitment (What does this mean, need to elaborate). Moreover, RT-PCR showed that SLPI mRNA levels in pathological tissues were noticeably elevated on average when compared with control rat eyes. Western blotting analysis also showed that SLPI protein expression in inflamed-infected eyes is up-regulated versus control samples.

## Conclusions

Our study indicates that SLPI may have an antimicrobial and antiprotease function, promoting up-regulation of local eye innate immunity and normal wound healing. These findings imply that SLPI contributes to host protection against inflammatory cell and enzyme mediated destruction of ocular tissues by modulating the immune response during infection and or inflammation.

